# New cases of dementia are rising in elderly populations in Wales, UK

**DOI:** 10.1016/j.jns.2023.120715

**Published:** 2023-06-19

**Authors:** Joshua Stevenson-Hoare, Ann-Kathrin Schalkamp, Cynthia Sandor, John Hardy, Valentina Escott-Price

**Affiliations:** 1 Department of Psychological Medicine and Clinical Neuroscience, Cardiff University, United Kingdom; 2 MRC Centre for Neuropsychiatric Genetics and Genomics, Cardiff University, United Kingdom; 3 UK Dementia Research Institute at Cardiff University, United Kingdom; 4 Department of Neurodegenerative Disease, UCL Institute of Neurology, United Kingdom; 5 UK Dementia Research Institute at UCL, London, United Kingdom

**Keywords:** Dementia, Alzheimer’s disease, vascular dementia, prevalence, incidence

## Abstract

Dementia is one of the most common diseases in elderly populations, and older populations are one of the fastest growing groups globally. Consequently, the number of people developing and living with dementia is likely to grow. Using longitudinal medical records from Wales, UK between 1999 and 2018, diagnoses of overall dementia and common subtypes were combined with demographic data to assess numbers of new and existing cases per year. Data extraction resulted in 161,186 diagnoses from 116,645 individuals. Mean age at diagnosis of dementia increased over this period, resulting in fewer younger people with the disease. New cases of dementia have risen, as has the number of people living with dementia. Individuals with dementia are also living longer, even accounting for their older age. This may present a challenge for healthcare systems as the number of elderly people living with dementia is expected to continue to grow.

## 1 Introduction

Dementia is primarily a disease of aging, with higher rates in older age groups. In the early 1980s it was described as “the silent epidemic” (Manton et al., 2005) and is now recognised by the World Health Organisation (WHO) as a public health priority (WHO, 2017). In the UK, 1.3% of the population is estimated to have dementia (Prince et al., 2014), ~800,000 people. In over-65s, dementia prevalence is 1 in 14 people, in over 80s, this rises to 1 in 6. In the UK over-65s are the fastest growing age group (Analytical Impact Team, 2021). As a result, it is reasonable to assume that the number of people with dementia is on the rise.

Work in recent years in western Europe has shown mixed results regarding dementia diagnoses over time. In Sweden, a stabilisation of number of people living with dementia suggests that there has been a decrease in the number of new cases (Qiu et al., 2013). In Germany there has been a fall in new cases, albeit coupled with increasing mortality (Doblhammer et al., 2015). In Denmark, however, the number of new dementia diagnoses have increased (Phung et al., 2010), which the authors attribute to better detection. One meta-analyses of European and North-American studies has suggested that there has been a fall in dementia incidence decade-on-decade (Wolters et al., 2020). However, in another meta-analysis this was not true for Japan (Roehr et al., 2018), a country with a very high proportion of the “oldest-old” (Yang et al., 2013). Additionally, these patterns differ between age groups (Tola-Arribas et al., 2013; Ziegler and Doblhammer, 2009; Ziegler-Graham et al., 2008), and between dementia subtypes (Bermejo-Pareja et al., 2008; Borroni et al., 2011; Perez et al., 2010). Consequently, it is important to look at dementia in homogenous populations as in broad populations true rates may be harder to detect.

The most common form of dementia is Alzheimer’s disease, accounting for 62% of cases (Prince et al., 2014). The second most common is vascular dementia at 17%. Other forms of dementia constitute single-digit percentages. There are also individuals who present with ‘undifferentiated’ dementia (Copeland et al., 1999), which is varied in symptoms and does not neatly fit into any established subtypes. Furthermore, some individuals present with multiple dementia subtypes. This may be due to overlap in diagnosis criteria, but in many cases these individuals have developed two dementia disorders concurrently, such as ‘AD-PD’ – Alzheimer’s disease and Parkinson’s disease (Feldman et al., 2014; Mrak and Griffin, 2007).

Incidence and prevalence are commonly used to assess disorder rates in a population. Incidence is the number of new diagnoses within a given time period. Prevalence is the number of active diagnoses at any given time point. Since dementia is degenerative and there is no treatment shown to reverse the course of disease, mortality rates need to be accounted for. As age is the strongest risk factor for the development of dementia, it is important to consider these statistics in different age groups. As these statistics only examine diagnosis rates, they cannot be used to directly assess dementia pathology and aetiology, e.g., whether individuals with pathological signs of subtype A are being clinically characterised as subtype B. However, with large enough populations, incidence and prevalence can be used to indirectly infer epidemiological trends in disease development.

Here, we study data on the Welsh population, sourced from the Secure Anonymised Information Linkage (SAIL) databank (Ford et al., 2009; Lyons et al., 2009). While the total population of Wales is smaller than that of the UK, at 3.1 million, dementia rates are representative of the UK as a whole. Recent estimates put the number of people living with dementia in Wales at 45,000 – or 1.4% (Phelps, 2015), only slightly higher than the UK average.

Our primary aim was to determine how dementia incidence and prevalence has changed in recent decades in Wales, and whether these changes could be explained by the increase in longevity in the UK population. We also explored the overlap between diagnoses of dementia subtypes. Finally, we quantified the rates of change in dementia diagnosis age, and survival time following diagnosis in the whole population and in subsets split by age in ten-year intervals from 30 to 99.

## 2 Materials and Methods

### 2.1 SAIL Databank and Datasets

The SAIL databank is a virtual platform that provides secure linkage between health-related datasets using anonymised IDs. Data on dementia diagnoses were collected from databases of hospital admission records including inpatients and day-cases, and records from general practitioners including investigations, symptoms, and referrals to specialist care. Demographic data were collected from a demographic dataset and primary care records. Details of the individual datatables queried are given in Supplemental Table 1.

Diagnosis of dementia was determined using ICD10 and NHS read codes (CTV2 and CTV3). ICD10 codes were identified by searching publicly available databases for named dementia subtypes and disorders containing ‘dementia’. NHS read codes were identified using the SAIL reference database (SAILREFV.READ_CD). Note that under the terms of the data access agreement, Dementia in HIV could not be analysed. A list of all ICD10 and NHS read codes used to identify dementia cases is provided in Supplemental Table 2.

Diagnosis tables were combined with demographic datasets to ensure that all individuals were resident in Wales at the time of diagnosis. Data were then merged into a unified dataset in R (R Core Team, 2021). Only data with a diagnosis date on/after January 1, 1999 and on/before December 31, 2018 were retained.

Age at diagnosis was not directly available in the dataset, so this was calculated using the date of diagnosis and date of birth. Early onset dementia can, rarely, occur in individuals in their 30s (Lücking et al., 2000; Mendez, 2006; Vieira et al., 2013). However, many diagnoses in the uncleaned dataset which occurred under age 30 were nonsensical, such as many cases of organic dementia (not related to alcohol or drug use) allegedly diagnosed under 20 years of age (N=4,456). As it is not possible to distinguish these errors from true diagnoses, and diagnosis of dementia is so rare below age 30, we excluded diagnoses under age 30.

Age at death was calculated for individuals with a date of death (DOD), and left blank for those without. Due to the length of time examined, DOD was available for most individuals in the dataset (N=93,965, 81.1%). Individuals without a DOD were presumed to be still living.

Deprivation indices used the Welsh Index of Multiple Deprivation (WIMD) (Welsh Government, 2015), a ranked score that identifies geographical areas of greatest and least deprivation across eight domains: income, employment, health, education, access to services, housing, community safety, and physical environment. Individuals are assigned a decile score from 1 (greatest deprivation) to 10 (least deprivation) based on their place of residence.

For incidence and prevalence calculations, only the first diagnosis of each dementia subtype was kept for each individual. As dementia diagnoses were included prior to 1999 for prevalence statistics, no washout period was used for overall inclusion (Roberts et al., 2015). For incidence we excluded any individuals with a recorded diagnosis of any dementia prior to 1^st^ January 1999. We also assessed co-occurrence of dementia subtypes, how often a person diagnosed with subtype A was also diagnosed with subtype B.

### 2.2 Analysis

#### 2.2.1 Incidence and prevalence calculation

Total incidence was the number of diagnoses that occurred within that year. Total prevalence was the number of living individuals with a diagnosis per year. To generate incidence and prevalence rates these numbers were scaled to per 1000 people, using statistics from WDSD on the number of individuals resident in Wales per year. For gender-specific calculations this was the number of males and females in the population. For prevalence we also included individuals with a date of diagnosis in 1998 or earlier, and whose date of death was not prior to 1^st^ January 1999, as they may still be alive in the study period.

#### 2.2.2 Statistical analysis

Linear regression models were fitted to assess whether the following changed depending on year of first diagnosis: a) age at diagnosis, b) survival time following diagnosis controlling for age at diagnosis, c) incidence rate per year, and d) prevalence rate per year. Since most literature reports rates for over-65-year olds, we also examined c) and d) in this subgroup. Comparison between males and females were made. All analyses were adjusted for gender and deprivation index.

Unless noted, results are reported without correction for multiple testing since the main findings were highly significant and survive Bonferroni correction for the number of tests performed in this study.

### 2.3 Ethical Approval

All data contained in SAIL has the permission from the relevant Caldicott Guardian or Data Protection Officer. SAIL-related projects are required to obtain Information Governance Review Panel (IGRP) approval.

## 3 Results

### 3.1 Dataset Demographics

#### 3.1.1 Dementia subtypes

**Figure 1:**
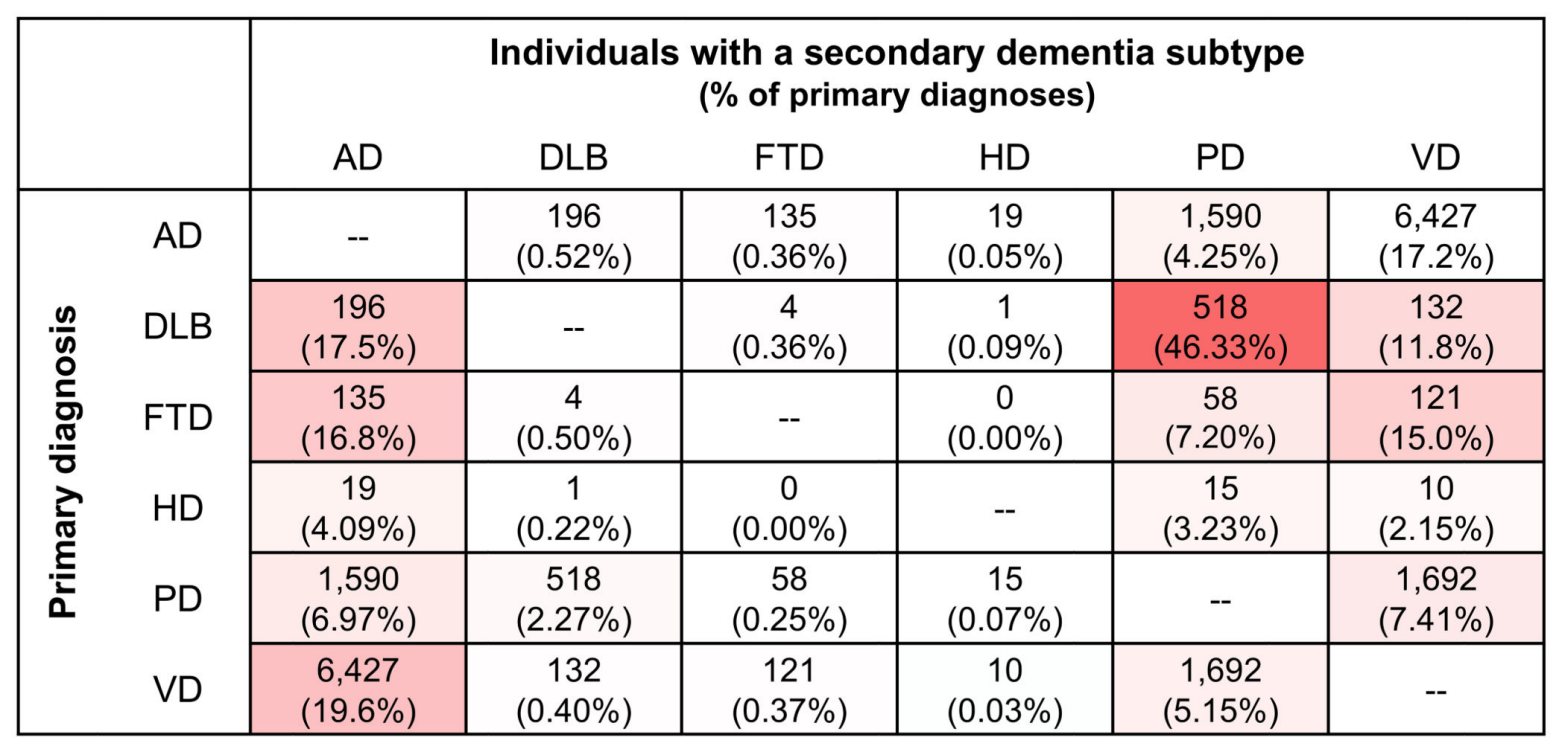
Co-occurrence of dementia subtypes, shown as the total and percentage of individuals with each primary diagnosis (rows) that also have a secondary diagnosis (columns). Colours represent percentage of diagnoses.

All instances of dementia in the datasets were extracted, and individuals were categorised into specific dementia subtypes where possible. These included Alzheimer’s disease (AD), dementia with Lewy bodies (DLB), frontotemporal dementia (FTD), Huntington’s disease (HD), Parkinson’s disease (PD), and vascular dementia (VD). For each individual, only the first instance of each diagnosis was kept. Individuals with a diagnosis of “Alcohol-induced” or “Drug-induced” dementia were removed from the dataset. This generated a dataset with 206,640 ‘unique’ diagnoses. Individuals could have multiple diagnoses provided they were different (e.g., AD and DLB). Figure 1 shows all co-occurrent dementia subtypes. The most common co-occurrent diagnosis was Alzheimer’s disease, being found alongside between 4-20% of other diagnoses. PD had the highest co-occurrence, with 46% of DLB diagnoses being shared with PD, however as these are both classified as “Lewy-body” dementias (Mrak and Griffin, 2007; Perez et al., 2010), this is unsurprising.

#### 3.1.2 Exclusion of non-dementia cases

Parkinson’s disease, while grouped with dementia disorders, does not necessarily cause dementia. Although there were no diagnoses of ‘Dementia in Parkinson’s disease’ (F02.3, ICD10), 33.1% of individuals with PD were diagnosed with at least one form of dementia separately, close to the estimated prevalence of dementia in PD of 30% (Aarsland and Kurz, 2010). These individuals were labelled as ‘Dementia in PD’ and kept in the dataset, and the PD category was removed from further analysis.

There were no diagnoses of ‘Dementia in Huntington’s disease’ (F02.1, ICD10) in the dataset. As with PD, individuals without an additional diagnosis were removed from the dataset, individuals with a dementia diagnosis (14.0%) were labelled as ‘Dementia in HD’, and HD was removed as a category. This left a dataset with 162,809 diagnoses from 116,645 unique individuals (Table 1).

Approximately half of diagnoses (77,359, 48.0%) were for specific dementia subtypes. The remaining diagnoses were non-specific dementia or dementia in other diseases (“Other dementias”). From the data it was not possible to verify whether an initial diagnosis was later superseded by a different diagnosis so we counted each initial instance per subtype. Of all individuals in the data, 75,294 (78.8%) were diagnosed with one dementia subtype only (Supplemental Table 3), excluding diagnoses that were for non-specific types, e.g. “senile dementia”. The most common diagnosis was AD, making up 22.9% of all diagnoses, followed by VD at 20.2% of diagnoses (Table 1). We note that the value for AD is lower than what has been established elsewhere (62% of dementia cases, Prince et al., 2014). This may be due to the high proportion of “other” dementia diagnoses, where a specific diagnosis is not provided. It is possible that the some of these undifferentiated dementia cases could be AD, but the tests necessary to differentiate AD (e.g., Amyloid PET scans) were not performed.

### 3.2 Age and survival time following dementia diagnosis

#### 3.2.1 Age at diagnosis

Linear regression models showed that age at diagnosis, increased between 1999 and 2018 for AD (B=0.24 [95% CI: 0.23, 0.24], *p*<10^-99^), Dementia in HD (B=0.80 [95% CI: 0.14, 1.23], *p*=0.009), Dementia in PD (B=0.30 [95% CI: 0.27, 0.34], *p*=7.86×10^-48^), DLB (B=0.17 [95% CI: 0.12, 0.21], *p*=0.001), VD (B=0.27 [95% CI: 0.24, 0.29], *p*<10^-99^), and overall dementia (B=0.25 [95% CI: 0.24, 0.25], *p*<10^-99^), controlling for gender and deprivation index (see also Table 1). For overall dementia this is equivalent to 3 months later each year. There was no significant change in age at diagnosis for FTD.

**Figure 2:**
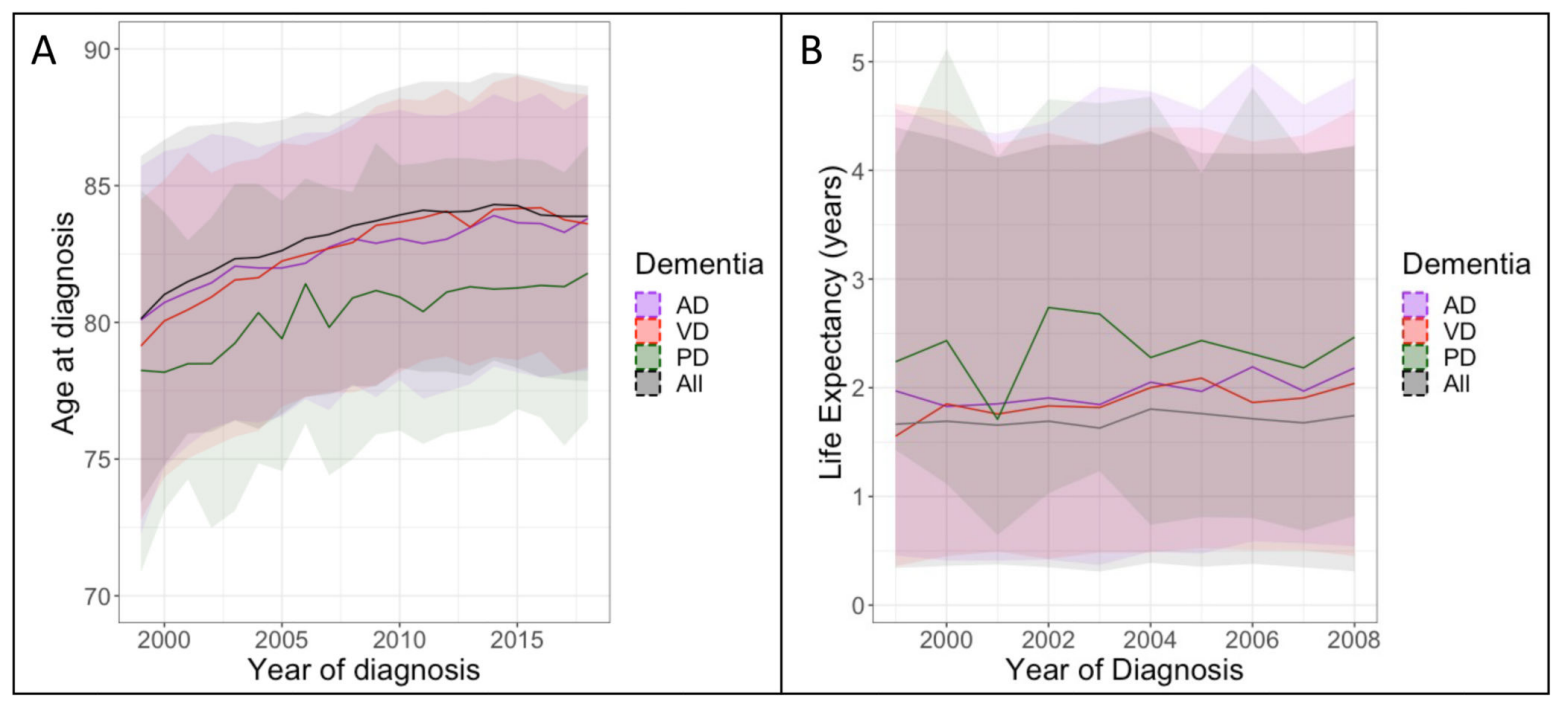
Median (A) age at diagnosis between 1999 and 2018 and (B) survival time following diagnosis between 1999 and 2008, for overall dementia, Alzheimer’s disease, vascular dementia, and Dementia in PD. Ribbons indicate 25% to 75% quartiles.

The change in age at diagnosis for AD, VD, Dementia in PD, and overall dementia are shown per year in Figure 2A (see Supplemental Table 4 for details). DLB and Dementia in HD are not plotted due to the small number of cases of these subtypes.

The change in age at diagnosis for overall dementia was not different between males and females (B=0.26, 0.24, n.s.), nor were there any gender differences for AD, Dementia in PD or VD.

#### 3.2.2 Survival time following diagnosis

Survival time following diagnosis was calculated as the time between date of diagnosis and date of death. Three-quarters of all deaths were less than five years after diagnosis. Additionally, 93% of all deaths were less than ten years following diagnosis. We therefore restricted analysis to individuals diagnosed at least a decade prior to the end of the study period (i.e., before December 31, 2008), and who died within ten years following diagnosis. This captured the survival time for over 90% of individuals, and prevented ceiling effects caused by dataset availability. Descriptive statistics for survival time are presented in Table 2.

Survival time after diagnosis increased between 1999 and 2008 for AD (B=0.06 [95% CI: 0.04, 0.06], *p*=2.12×10^-14^), VD (B=0.06 [95% CI: 0.05, 0.08], *p*=1.94×10^-14^), and overall dementia (B=0.04 [95% CI: 0.03, 0.04], *p*=2.24×10^-24^), controlling for gender, age at diagnosis, and deprivation index. For overall dementia this is an increase in survival time of approximately 2 weeks per year (AD and VD: 3 weeks). Survival time did not significantly change for Dementia in PD (*p*=0.563). The changes in survival time for AD, VD, and overall dementia are shown in Figure 2B, detailed results are shown in Supplemental Table 5. We did not calculate linear regression models for DLB, FTD, or Dementia in HD due to their small sample sizes. The change in survival time for was not significantly different between males and females for overall dementia, AD, or VD.

### 3.3 Incidence

**Figure 3:**
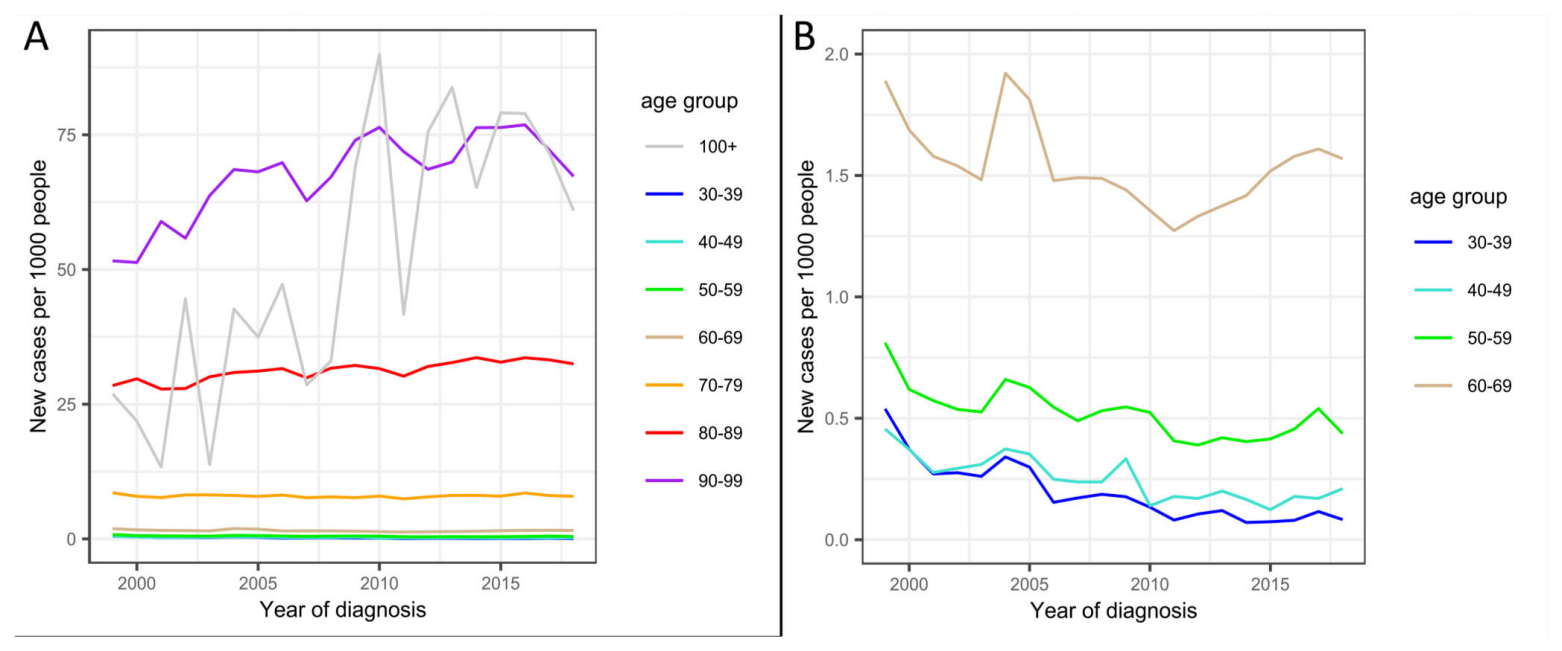
Incidence of overall dementia per 1000 people between 1999 and 2018 for (A) all age categories, and (B) zoomed version with y-axis between 0 and 2, to show data for individuals under 70.

New cases of dementia have risen between 1999 and 2018 for overall dementia (B=0.059 [95% CI: 0.044, 0.075], *p*=1.81×10^-10^), adjusting for age demographics. Incidence rates for overall dementia are shown in Figure 3 split by age in ten-year intervals from 30 to 90+, and for AD and VD in Supplemental Figure 1. Rates for DLB, FTD, and Dementia in PD and HD were not calculated due to small sample sizes. Over 100s were grouped with over 90s, due to the small sample size of this group.

Incidence rates for overall dementia decreased between 1999 and 2018 for under 60s, did not significantly change for 60-79s (accounting for multiple testing), and increased for over 80s. Incidence changes were greatest in over 90s, with an increase of 0.8 diagnoses per thousand people, per year. These results are presented in Supplemental Table 6. AD and VD showed the same pattern of results, shown in Supplemental Tables 7-8.

Comparison of incidence rates between males and females, adjusted for gender balance in the population, did not show any significant difference in the number of new overall dementia cases per year, accounting for age and year of diagnosis (*p*=0.889). There were significantly more new cases in females than males in AD and VD (*p*=1.94×10^-11^, *p*=0.004, respectively).

### 3.4 Prevalence

**Figure 4:**
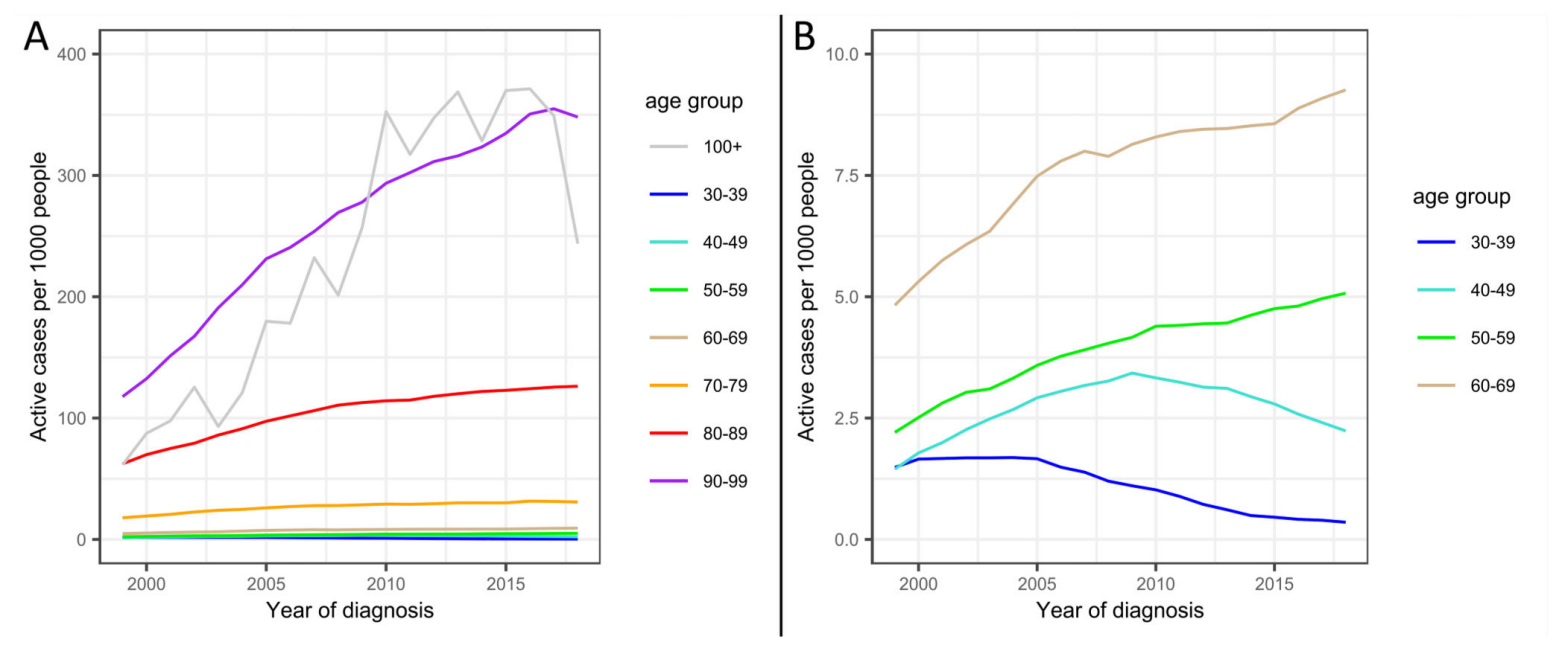
Prevalence of overall dementia per 1000 people between 1999 and 2018 for (A) all age categories, and (B) zoomed version with y-axis between 0 and 10, to show data for individuals under 70.

The number of people living with dementia has risen between 1999 and 2018 for overall dementia (B=0.59 [95% CI: 0.43, 0.79], *p*=1.81×10^-29^), adjusting for age category as a random effect in a mixed model analysis. Prevalence rates for overall dementia split by age are shown in Figure 4. Prevalence rates for AD and VD are plotted in Supplemental Figure 2. Prevalence rates were not calculated for DLB, FTD, or Dementia in PD and HD due to small sample sizes.

Prevalence rates for overall dementia significantly decreased for under 50s, did not change for 60-69s, and significantly increased for over 70s. Prevalence rate changes were greatest in over 90s, with an additional 12.6 individuals per thousand people per year. The results of this model are presented in Supplemental Table 9. For both AD and VD, prevalence fell in under 40s, was stable for 40-49s, and rose for over 50s (Supplemental Tables 10-11).

Comparison of prevalence between males and females, adjusted for gender balance, showed a nominally significantly greater number of females living with overall dementia than males, accounting for age and year of diagnosis (*p*=0.019). Similar to incidence, there were significantly more females than males living with AD (*p*=2.38×10^-6^), and with VD (*p*=0.011).

### 3.5 Comparison of early-onset and late-onset dementias

Individuals were split into “early-onset” and “late-onset” dementia depending on whether their first diagnosis was prior to or after age 65, respectively. In AD, VD, dementia in PD, and DLB, more than 90% of cases were late-onset. Early-onset dementia made up 45.9% of Dementia in HD cases, and 24.4% of FTD cases. Full results are given in Supplemental Tables 12 and 13.

Dementia incidence decreased per year for early onset dementia overall (p=1.07×10^-5^), as well as for AD (*p*=1.28×10^-6^), Dementia in PD (*p*=4.88×10^-8^), and VD (*p*=2.82×10^-5^). Early onset DLB did not significantly change (*p*=0.218), and early onset FTD increased (*p*=7.62×10^-5^). For late onset dementia, overall incidence increased (*p*=0.010), as did incidence for AD (*p*=8.50×10^-5^), DLB (*p*=9.84×10^-12^), FTD (*p*=1.42×10^-5^), and VD (*p*=1.24×10^-5^). Late onset PD incidence decreased (*p*=3.55×10^-6^). Finally, there were too few cases to calculate linear models for Dementia in HD.

Prevalence increased for both early and late onset overall dementia (*p*=1.38×10^-11^, *p*=1.52×10^-8^), AD (*p*=1.37×10^-10^, *p*=6.78×10^-14^), DLB (*p*=1.57×10^-10^, *p*=6.97×10^-19^), FTD (*p*=3.25×10^-16^, *p*=8.52×10^-10^), and VD (*p*=7.07×10^-11^, *p*=1.52×10^-8^). There was no change in prevalence for Dementia in PD (*p*=0.108, *p*=0.165).

These findings match what has been shown above using ten-year age brackets, and demonstrates that using broader categories such as early/late onset still shows the general trend for later diagnoses of dementia becoming more common.

## 4 Discussion

Medical records from the SAIL databank were used to examine the incidence and prevalence of dementia in Wales, UK between 1999 and 2018. The majority of dementia cases in Wales in the study period were diagnoses of Alzheimer’s disease or vascular dementia, with a large proportion of non-specific dementia diagnoses. The remainder were split between Lewy-body dementias (including Parkinson’s disease), frontotemporal dementia, and Huntington’s disease.

Our analyses showed that incidence and prevalence rates of overall dementia have increased between 1999 and 2018. When broken down by age categories to understand the dynamics of dementia with age, incidence rates increased in older people (above 70), and decreased in younger people (below 60). Similar patterns were observed for AD and VD. Dementia prevalence rates significantly decreased only in under 40s, and increased in over 50s. We did not observe any difference in incidence between males and females for overall dementia, but we did find greater prevalence for females compared to males. There were no significant differences in the change in incidence or prevalence rates over time between males and females, with a trend of a slightly greater number of females living with overall dementia than males. Additionally, we did not find any differences in age of diagnosis or survival time following diagnosis between males and females.

The total number of people living with dementia in Wales at the end of 2018, according to our dataset, was 40,455, lower than the prediction from (Wittenberg et al., 2019) for 2018 of 46,800. However, the SAIL databank does not have total coverage for Wales. The WLGP dataset contains records from 82.5% of GP practices, with coverage varying between 43.75% and 95.38% across health boards (Analytical Services Public, 2022). Note, though, that all areas except for Powys LHB had over 75% coverage. When upscaled to the total population in Wales, incidence and prevalence numbers more closely match other’s estimates.

Published estimates of incidence and prevalence for dementia typically either discuss rates for the total population, or for over-65s only, therefore we also calculated these values. Wittenburg et al. (Wittenberg et al., 2019) estimate dementia prevalence rate in Wales for 2019 as 15.1 per 1000 for the total population and 70.7 per 1000 for over-65s. Our results are similar (16.1 per 1000 for total population, and 59.9 per 1000 for over-65s), although our data only record up to 2018. For dementia incidence we can compare the results from our dataset to those published in Matthews et al. (Matthews et al., 2016) in 2016. They estimate the number of new cases in the total population as 3.2 per 1000, and 17.8 per 1000 for over-65s. Our results for 2016 are similar, with 4.5 per 1000 in the total population, and 13.4 per 1000 for over-65s.

Given that the elderly population in the UK is rising (Analytical Impact Team, 2021), it is also of interest to predict how dementia incidence and prevalence may continue into the future. For example, some groups have projected as high as a three-fold increase in dementia prevalence (GBD 2019 Dementia Forecasting Collaborators, 2022) between 2019 and 2050. If a linear increase continues until 2050, our data would suggest an all-cause dementia incidence rate of 6.5 per 1000 people in the total population, and 23.2 per 1000 in over-65s. For prevalence, with a linear trend, our data suggest an all-cause dementia prevalence of 34.9 per 1000 in the total population and 264 per 1000 in over 65s. However, a continuing linear increase in incidence and prevalence is unlikely to be the case, as we also saw an increase in the age of diagnosis and survival time after diagnosis. There is also the possibility of the oldest age-groups plateauing (Barbi et al., 2018), in which case these trends in incidence and prevalence may also plateau. Additionally, the development of medical and lifestyle interventions for both pre-onset and post-onset of dementia are likely to have an impact on these rates.

As the increase in prevalence rate may be attributable to longevity, we tested and confirmed that that 1) the age at diagnosis for dementia over the study period has increased, and 2) survival time following diagnosis of people living with dementia increased controlling for age at diagnosis (as later life diagnoses may naturally result in shorter survival time). Thus, our results suggest that not only are individuals developing dementia later in life, they are also living with the disease for longer. This may be attributable to lifestyle delaying onset and better healthcare both before and after disease onset. Alternatively, this may be partly attributable to changes in public attitudes to dementia, such as reduced stigma for seeking help. This change may also indicate diagnosis being made earlier in the progression of the disease, so duration following diagnosis appears to be longer. However, as the actual age at diagnosis has increase over the last decades, earlier detection is unlikely to fully explain the extending of survival time.

Our estimates of incidence and prevalence for the total Welsh population are larger than other published sources, possibly due to the inclusion of a wider range of dementia diagnoses, whereas other sources may be limited to primary diagnoses. However, our results for the over-65 population show lower incidence and prevalence than other sources. This may be due to under-diagnosis of dementia in older individuals (Prince et al., 2014). For example, Alzheimer’s Research UK estimate that 47% of dementia cases in Wales are not officially diagnosed (Dementia Statistics Hub, 2021). As our dataset uses medical records, this will not reflect individuals who have developed dementia but not sought diagnosis.

A potential limitation of our study is that the datasets we used covered a period of twenty years, during which time diagnosis criteria has changed for some disorders, e.g., (“Alzheimer’s Disease Diagnostic Guidelines,” n.d.; Postuma et al., 2016). Such changes can also take time to be applied universally. For example, in 2011, mortality coding practice for dementia was changed for GPs (Hayat et al., 2022), which altered the cause of death put on certificates. In 2012, a “coding clean-up” exercise was launched in London, UK (Russell et al., 2013), which encouraged the back-dating of dementia diagnosis codes in dementia patients without appropriate labels. The authors estimated this could result in as many as 70,000 additional dementia cases being registered that were otherwise unknown to the system. Our data were last updated in 2019, after the launch of this exercise. However, although this has since been taken up by a number of English NHS groups (London Dementia Clinical Network, 2017), it is unclear whether this has spread as far as the Welsh NHS service (*Gwasanaeth Iechyd Gwladol Cymru*), which is distinct from the English NHS.

Analysis of the choice of codes made by the diagnosing individuals showed that, in disorders where multiple code prefixes were available (e.g., Alzheimer’s disease is labelled as both F00 and G30 in ICD10), there was no time-related element to the choice of code used. I.e., for Alzheimer’s disease, F00 and G30 were used equally across the study period and there were no times when either code was the predominant choice by clinicians. This supports the notion that any substantive changes to dementia coding best practice are not universally uptaken.

Finally, we do not have information on the professional identity of diagnosing clinicians, i.e. general practitioner, neurologist, or psychologist. Meta-analyses have shown that specialists such as radiologists or experienced clinicians are typically accurate in differentiating subtypes (Klöppel et al., 2008; Lopez et al., 2011). However, general practitioners often perform more poorly, particularly for patients in early stages of dementia or cognitive impairment (Mitchell et al., 2011; van den Dungen et al., 2012), especially for less common forms of dementia (Mendez, 2006). We found substantial overlaps between dementia subtypes, particularly with AD and VD. These are the most common dementia subtypes (Prince et al., 2014), so this may reflect initial misdiagnosis favouring more common subtypes. This has an important impact for, e.g., genome-wide association studies, as misdiagnosed individuals can introduce incorrect interpretations of genetic findings.

## 5 Conclusions

Medical records for Wales show that new diagnoses of dementia have risen in older people between 1999 and 2018, and that overall numbers of diagnosed dementia cases have increased in this same period. This is linked to the aging and greater survival time following diagnosis. Our results show that these rates are likely to continue increasing for the foreseeable future, while falling for younger age groups. By studying trends from the recent past we are able to track the “silent epidemic” of dementia, and therefore better manage our response going forward fighting this devastating disease.

## Supplementary Material

Supplemental

## Figures and Tables

**Table 1:** Descriptive statistics including N available for age at diagnosis and age at death split by disorder, collapsed across 1999 to 2018. Percentages in brackets are the percentage of all diagnoses (N=162,809) that were for that type.

Disorder	N diagnosed	N deceased	Gender % (M / F)	Mean age at diagnosis (SD)	Mean age at death (SD)
AD	37,430 (22.9%)	29,025 (18.0%)	36.6 / 63.4	81.2 (9.61)	85.1 (7.61)
DLB	1,115 (0.68%)	842 (0.52%)	59.7 / 40.3	78.1 (8.09)	81.7 (7.64)
FTD	805 (0.49%)	617 (0.38%)	47.5 / 52.5	74.0 (12.3)	78.7 (10.8)
Dementia in HD	68 (0.04%)	52 (0.03%)	44.1 / 55.9	69.1 (13.8)	75.6 (12.2)
Dementia in PD	6,754 (4.15%)	5,632 (3.49%)	57.3 / 42.7	77.7 (9.48)	82.8 (7.69)
VD	32,810 (20.2%)	27,014 (16.8%)	43.2 / 56.8	81.8 (8.61)	85.2 (7.27)
Other dementias	83,827 (51.2%)	69,797 (43.3%)	38.2 / 61.8	81.8 (10.9)	85.5 (8.79)
All individuals	116,645	93,565	39.1 / 60.9	81.0 (10.9)	82.6 (9.00)

**Table 2:** Descriptive statistics including N available survival time after diagnosis split by disorder, collapsed across 1999 to 2008.

Disorder	N individuals	Gender % M / F	Median survival (IQR)
AD	14,271	34.9 / 65.1	1.81 (3.71)
DLB	255	55.7 / 44.3	3.13 (3.93)
FTD	181	50.3 / 49.7	2.15 (4.67)
Dementia in HD	30	40.0 / 60.0	4.29 (4.67)
Dementia in PD	2,882	54.8 / 45.2	3.04 (4.82)
VD	11,535	44.2 / 55.8	1.84 (3.62)
Other dementias	31,146	36.3 / 63.7	1.44 (3.40)
All individuals	45,617	37.7 / 62.3	1.80 (3.80)
