# Supplementary material for: New cases of dementia are rising in elderly populations in Wales, UK
Source: J Neurol Sci. Author manuscript; Available in PMC 2024 Jan 29. (PMC7615574; doi:10.1016/j.jns.2023.120715)

# Supplemental Materials

Supplemental Table 1. Queried data tables from the SAIL databank.

| **Database** | **Table** | **Rows** | **Description** |
| --- | --- | --- | --- |
| PEDW | DIAG_20191213 | 81,918,997 | Diagnosis of disease or disorder |
| PEDW | SPELL_20191213 | 19,770,608 | Period of time patient is affected with disease or disorder |
| WDSD | AR_PERS_20191213 | 4,765,177 | Demographic information |
| WLGP | CLEAN_REG_GP_MEDIAN_20200127 | 4,410,779 | Demographic information |
| WLGP | EVENT_ALF_CLEANSED_20200127 | 2,998,833,329 | Diagnosis and/or treatment of disease or disorder |

Supplemental Table 2. ICD 10 and NHS read codes for dementia-related disorders and their labels.

| **Disorder** | **ICD 10 code(s)** | **NHS read code(s)** |
| --- | --- | --- |
| Alcohol-induced dementia |  | E01 |
| Alzheimer's disease | F00, G30 | Eu00, F110 |
| Drug-induced dementia |  | E02 |
| Frontotemporal dementia | F02.0, G31.0 | Eu020, F111 |
| Huntington's disease | F02.2, G10 | Eu022 |
| Lewy-body dementia | G31.83 | Eu025, F116 |
| Parkinson's disease | F02.3, G20 | Eu023, F12 |
| Presenile dementia |  | E001 |
| Senile dementia |  | E000, E002, E003 |
| Vascular dementia | F01 | E004, Eu01 |
| Unspecified dementia, dementia in other diseases | F02.1, F02.4, F02.8, F03, G31 | E04, Eu02 |

Supplemental Table 3. Number of individuals diagnosed with a single subtype only (% of individuals with that diagnosis).

| **Disorder** | **Individuals** |
| --- | --- |
| Alzheimer’s disease | 29,576 (79.0%) |
| Dementia with Lewy-bodies | 435 (38.9%) |
| Frontotemporal dementia | 538 (66.8%) |
| Huntington’s disease | 431 (92.7%) |
| Parkinson’s disease | 19,417 (85.1%) |
| Vascular dementia | 24,897 (75.8%) |

Supplemental Table 4. Results of linear models predicting age at diagnosis from year of diagnosis, controlling for sex.

| **Dementia Subtype** | **Beta** | ***P* value** | **R2** |
| --- | --- | --- | --- |
| AD | 0.244 | < 10^-99 †^ | .033 |
| DLB | 0.167 | .001 | .039 |
| FTD | 0.145 | .068 | .011 |
| Dementia in HD | 0.798 | .009 | .129 |
| Dementia in PD | 0.301 | 7.78×10^-48^ | .035 |
| VD | 0.273 | < 10^-99 †^ | .048 |
| All data | 0.245 | < 10^-99 †^ | .041 |

^†^ It was not possible to retrieve this *P* value as it was below the minimum reportable threshold in R.

Supplemental Table 5. Results of linear models predicting survival time after diagnosis between 1999 and 2008 from year of diagnosis, controlling for age at diagnosis and sex.

| **Dementia Subtype** | **N Deceased** | **Beta** | ***P* value** | **R2** |
| --- | --- | --- | --- | --- |
| AD | 14,271 | 0.056 | 2.12×10^-14^ | .047 |
| Dementia in PD | 2,882 | 0.011 | .563 | .063 |
| VD | 11,535 | 0.064 | 1.94×10^-14^ | .055 |
| All data | 45,617 | 0.042 | 2.24×10^-24^ | .064 |

Supplemental Table 6. Results of linear and mixed-effects models predicting incidence of dementia per 1000 people, predicted by year of diagnosis.

| **Age Category** | **N Diagnoses** | **Beta** | ***P* value** | **R2** |
| --- | --- | --- | --- | --- |
| 30 – 39 | 1,253 | -0.018 | 4.61 × 10^-7^ | .765 |
| 40 – 49 | 1,605 | -0.013 | 1.11 × 10^-5^ | .667 |
| 50 – 59 | 3,134 | -0.013 | 1.48 × 10^-4^ | .560 |
| 60 – 69 | 7,705 | -0.014 | .041 | .212 |
| 70 – 79 | 28,192 | -0.003 | .745 | .006 |
| 80 – 89 | 53,686 | 0.261 | 8.89 × 10^-7^ | .747 |
| 90+ | 19,445 | 0.838 | 1.12 × 10^-4^ | .573 |
| All Ages | 116,414 | 0.059 | 1.81 × 10^-10^ | .901 |

Supplemental Table 7. Results of linear and mixed-effects models predicting incidence of Alzheimer's disease per 1000 people, predicted by year of diagnosis.

| **Age Category** | **N Diagnoses** | **Beta** | ***P* value** | **R2** |
| --- | --- | --- | --- | --- |
| 30 – 39 | 298 | -0.007 | 2.45 × 10^-6^ | .718 |
| 40 – 49 | 274 | -0.005 | 6.35 × 10^-7^ | .756 |
| 50 – 59 | 732 | -0.006 | 2.63 × 10^-6^ | .716 |
| 60 – 69 | 2,260 | -0.006 | .026 | .246 |
| 70 – 79 | 10,111 | 0.018 | .068 | .173 |
| 80 – 89 | 18,590 | 0.197 | 1.70 × 10^-4^ | .553 |
| 90+ | 5,133 | 0.399 | 1.51 × 10^-4^ | .559 |
| All Ages | 37,407 | 0.028 | 2.45 × 10^-5^ | .636 |

Supplemental Table 8. Results of linear and mixed-effects models predicting incidence of vascular dementia per 1000 people, predicted by year of diagnosis.

| **Age Category** | **N Diagnoses** | **Beta** | ***P* value** | **R2** |
| --- | --- | --- | --- | --- |
| 30 – 39 | 165 | -0.003 | 3.60 × 10^-6^ | .706 |
| 40 – 49 | 153 | -0.003 | 5.35 × 10^-6^ | .693 |
| 50 – 59 | 391 | -0.002 | .002 | .416 |
| 60 – 69 | 1,880 | -0.002 | .286 | .063 |
| 70 – 79 | 8,659 | 0.035 | .002 | .436 |
| 80 – 89 | 16,202 | 0.326 | 6.94 × 10^-7^ | .754 |
| 90+ | 4,754 | 0.831 | 9.08 × 10^-9^ | .847 |
| All Ages | 32,158 | 0.041 | 4.34 × 10^-9^ | .859 |

Supplemental Table 9. Results of linear and mixed-effects models predicting prevalence of dementia per 1000 people, predicted by year of diagnosis.

| **Age Category** | **N Case-years** | **Beta** | ***P* value** | **R2** |
| --- | --- | --- | --- | --- |
| 30 – 39 | 7,036 | -0.084 | 6.55 × 10^-11^ | .911 |
| 40 – 49 | 17,779 | 0.040 | .059 | .184 |
| 50 – 59 | 23,885 | 0.141 | 5.54 × 10^-14^ | .960 |
| 60 – 69 | 39,063 | 0.208 | 4.31 × 10^-10^ | .891 |
| 70 – 79 | 96,128 | 0.651 | 7.90 × 10^-10^ | .883 |
| 80 – 89 | 181,850 | 3.27 | 2.04 × 10^-11^ | .922 |
| 90+ | 107,046 | 18.06 | 2.73 × 10^-15^ | .971 |
| All Ages | 497,410 | 0.587 | 1.81 × 10^-29^ | .999 |

Supplemental Table 10. Results of linear and mixed-effects models predicting prevalence of Alzheimer's disease per 1000 people, predicted by year of diagnosis.

| **Age Category** | **N Case-years** | **Beta** | ***P* value** | **R2** |
| --- | --- | --- | --- | --- |
| 30 – 39 | 1,824 | -0.033 | 3.42 × 10^-12^ | .936 |
| 40 – 49 | 4,849 | -0.003 | .724 | .007 |
| 50 – 59 | 6,380 | 0.032 | 3.89 × 10^-12^ | .935 |
| 60 – 69 | 11,328 | 0.042 | 4.21 × 10^-9^ | .860 |
| 70 – 79 | 33,656 | 0.217 | 1.40 × 10^-12^ | .942 |
| 80 – 89 | 65,202 | 1.34 | 5.98 × 10^-14^ | .959 |
| 90+ | 23,962 | 4.23 | 1.04 × 10^-15^ | .974 |
| All Ages | 165,100 | 0.201 | 1.38 × 10^-12^ | .973 |

Supplemental Table 11. Results of linear and mixed-effects models predicting prevalence of vascular dementia per 1000 people, predicted by year of diagnosis.

| **Age Category** | **N Case-years** | **Beta** | ***P* value** | **R2** |
| --- | --- | --- | --- | --- |
| 30 – 39 | 1,109 | -0.013 | 8.72 × 10^-8^ | .804 |
| 40 – 49 | 2,341 | 0.003 | .147 | .113 |
| 50 – 59 | 3,106 | 0.020 | 2.29 × 10^-8^ | .831 |
| 60 – 69 | 7,397 | 0.033 | 7.73 × 10^-7^ | .751 |
| 70 – 79 | 26,980 | 0.269 | 4.04 × 10^-7^ | .768 |
| 80 – 89 | 52,485 | 1.67 | 3.01 × 10^-10^ | .895 |
| 90+ | 18,792 | 5.23 | 3.23 × 10^-17^ | .982 |
| All Ages | 125,474 | 0.197 | 1.85 × 10^-19^ | .990 |

Supplemental Table 12. Results of linear models predicting incidence of early onset (before age 65) and late onset dementias scaled to the population, predicted by year of diagnosis.

|  | **Early Onset** | | | **Late Onset** | | |
| --- | --- | --- | --- | --- | --- | --- |
| **Dementia Subtype** | **N cases** | **Beta** | ***P* value** | **N cases** | **Beta** | ***P* value** |
| AD | 2,033 | -0.005 | 1.28×10^-6^ | 35,397 | 0.065 | 8.50×10^-5^ |
| DLB | 72 | 9.60×10^-5^ | 0.218 | 1,043 | 0.010 | 9.84×10^-12^ |
| FTD | 191 | 5.79×10^-4^ | 7.62×10^-5^ | 614 | 0.005 | 1.42×10^-5^ |
| Dementia in PD | 583 | -0.002 | 4.88×10^-8^ | 6,171 | -0.022 | 3.55×10^-6^ |
| VD | 1,297 | -0.003 | 2.82×10^-5^ | 31,513 | 0.102 | 1.24×10^-5^ |
| All data | 8,922 | -0.015 | 1.07×10^-5^ | 107,723 | 0.043 | 0.010 |

Supplemental Table 13. Results of linear models predicting prevalence of early onset (before age 65) and late onset dementias, predicted by year of diagnosis.

|  | **Early Onset** | | | **Late Onset** | | |
| --- | --- | --- | --- | --- | --- | --- |
| **Dementia Subtype** | **N case-years** | **Beta** | ***P* value** | **N case-years** | **Beta** | ***P* value** |
| AD | 26,300 | 0.038 | 1.37×10^-10^ | 138,787 | 0.556 | 6.78×10^-14^ |
| DLB | 465 | 0.002 | 1.57×10^-10^ | 3,738 | 0.045 | 6.97×10^-19^ |
| FTD | 1,206 | 0.005 | 3.25×10^-16^ | 2,181 | 0.013 | 8.52×10^-10^ |
| Dementia in PD | 10,740 | -0.003 | 0.108 | 37,281 | -0.035 | 0.165 |
| VD | 15,000 | 0.027 | 7.07×10^-11^ | 116,323 | 0.657 | 2.06×10^-9^ |
| All data | 103,851 | 0.171 | 1.38×10^-11^ | 425,424 | 1.332 | 1.52×10^-8^ |

Supplemental Figure 1. Incidence of dementia per 1000 people between 1999 and 2018 for all age categories in (A) Alzheimer’s disease and (B) Vascular dementia. Incidence in over 100s is not plotted due to low numbers of cases. Graphs (C,D) are zoomed version of (A,B) with y-axis between 0 and 0.8, to show data for individuals under 70.


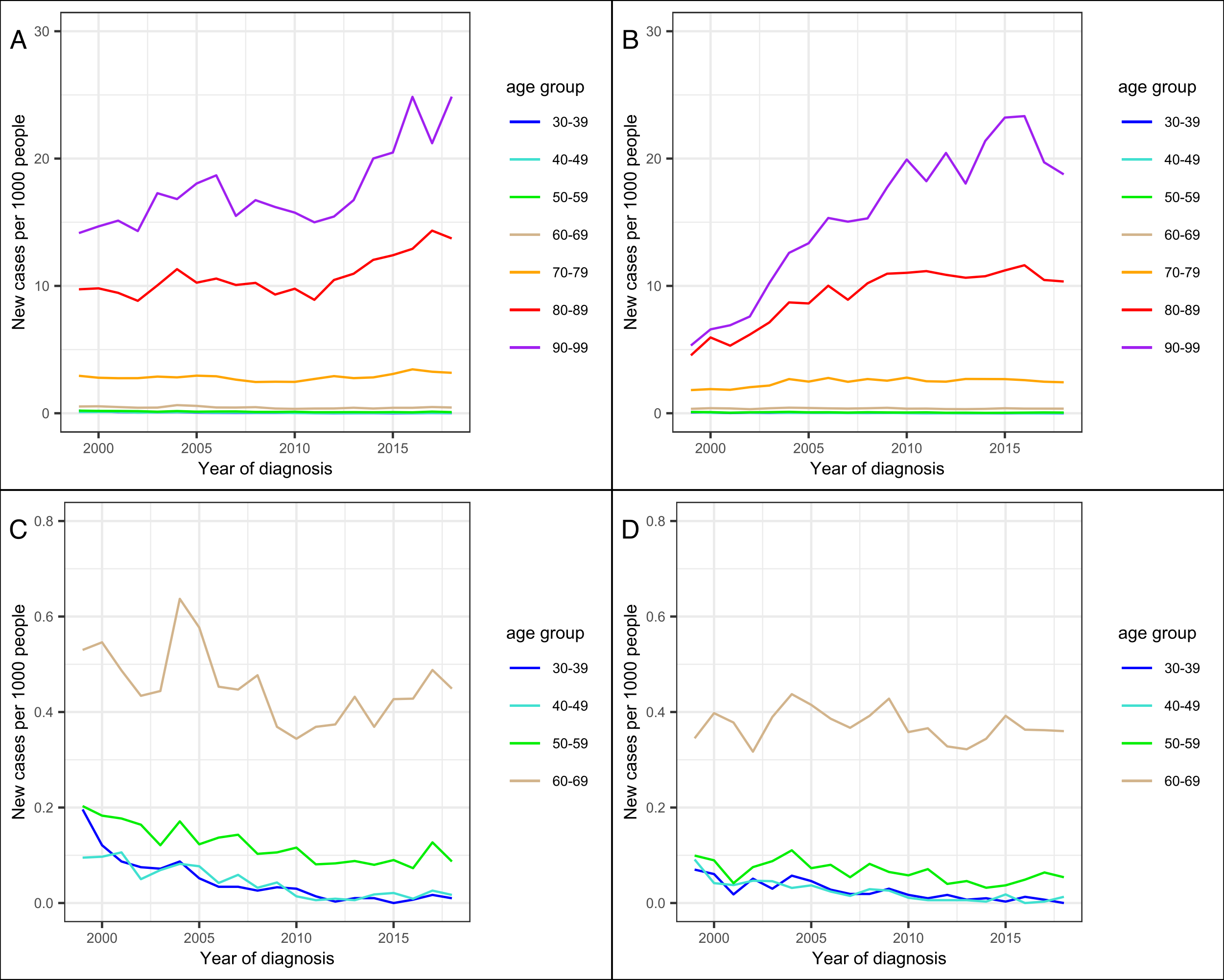


Supplemental Figure 2. Prevalence of dementia per 1000 people between 1999 and 2018 for all age categories in (A) Alzheimer’s disease, and (B) Vascular dementia. Prevalence in over 100s is not plotted due to low numbers of cases. Graphs (C,D) are zoomed version of (A,B) with y-axis between 0 and 3, to show data for individuals under 70.


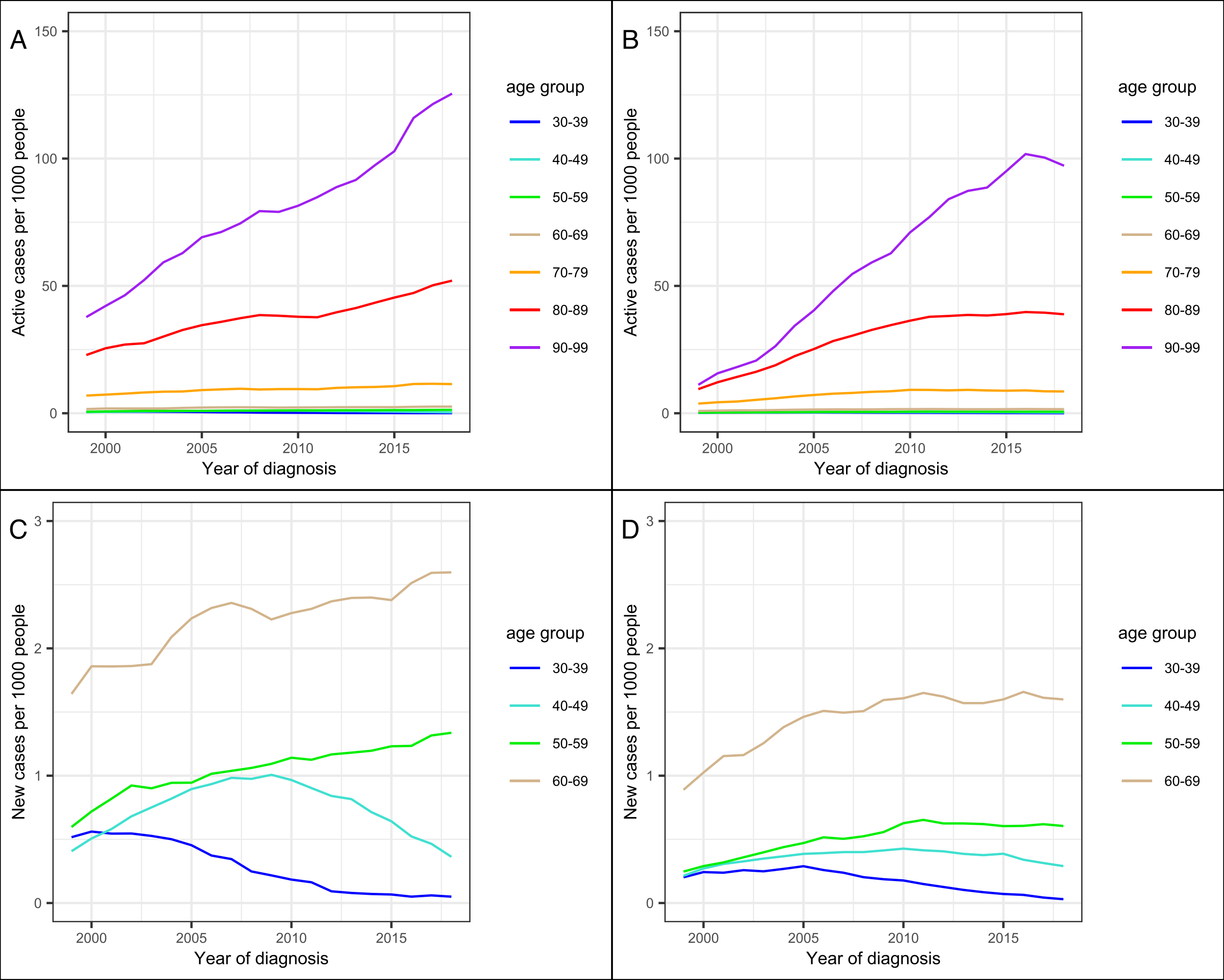

Supplement: Supplemental [file EMS193579-supplement-Supplemental.docx]
